# What are Mental Disorders? Exploring the Role of Culture in the Harmful Dysfunction Approach

**DOI:** 10.1007/s12124-024-09837-9

**Published:** 2024-04-03

**Authors:** Svend Brinkmann

**Affiliations:** Aalborg University, Teglgårds Plads 1, Aalborg, 9000 Denmark

**Keywords:** Mental Disorder, Harmful Dysfunction, Culture, Naturalism, Evolution, Mentalism

## Abstract

A shared problem in psychology, psychiatry, and philosophy is how to define mental disorders. Various theories have been proposed, ranging from naturalism to social constructionism. In this article, I first briefly introduce the current landscape of such theories, before concentrating on one of the most influential approaches today: The harmful dysfunction theory developed by Jerome Wakefield. It claims that mental disorders are hybrid phenomena since they have a natural basis in dysfunctional mental mechanisms, but also a cultural component in the harm experienced by human beings. Although the theory is well thought through, I will raise a critical question: Is it possible to isolate mental mechanisms as naturally evolved from cultural factors? I will argue that it is not, but that the theory could still be helpful in an understanding of mental disorders, albeit on a new footing that does not operate with a natural and a cultural component as two separate factors. I argue that we need to develop a “naturecultural” approach to psychopathology that avoids mentalism, based on the fact that human beings are irreducibly persons.

## Introduction

A shared problem in psychology, psychiatry, and philosophy is how to define mental disorders. Some scholars have rejected the term entirely, claiming that talk of mental illness and disorder simply represents an illegitimate pathologization of unwanted behaviors (this was the thesis of anti-psychiatrists such as Szasz, 1961, who preferred to talk about “problems in living”), but most researchers and practitioners in the field of mental health find the term meaningful, but hard to define.^1^ For what is the exact difference between the experience of suffering in one’s life (e.g. because of hardship related to unemployment, divorce etc.) and suffering from a mental disorder?

Various theories have been proposed to answer this question, ranging from naturalism to social constructionism. Below, I shall first briefly introduce the current landscape of such theories, before concentrating on one of the most influential approaches today: The harmful dysfunction theory developed by Wakefield (1992). This theory claims that mental disorders are hybrid phenomena since they have a natural basis in dysfunctional mental mechanisms with an evolutionary history, but also a cultural component in the harm experienced by human beings. Although the theory is well thought through, I will confront it with two overarching critical questions: Is it possible to isolate mental mechanisms as naturally evolved from cultural factors? And is it even meaningful to talk about “mental mechanisms” in the first place? I will argue that it is not, but that the theory could still be helpful in an understanding of mental disorders, albeit on a new non-mentalist footing that does not operate with a natural and a cultural component as two separate factors. I argue that we need to develop a “naturecultural” approach to mental life and its disorders, based on the fact that human beings are irreducibly persons, where dysfunctions are at once natural and cultural, and I will explore whether this can be compatible with the main structure of Wakefield’s theory.

### A Landscape of Theories of Mental Disorder

If we bracket those radical approaches to mental disorder that are outright anti-psychiatric (e.g. the aforementioned Szasz, 1961), we may distinguish between naturalist theories, on the one hand, and theories that are cultural or constructionist on the other. The latter group of theories can also be called normativist, since they are based on the view that judgments about disorder are ultimately normative value judgments (De Block et al., 2023). Mental disorders are wide-ranging and include schizophrenia, bipolar disorder, anxiety, depression, autism, ADHD and much more, and it is particularly challenging to figure out what (if anything) legitimizes uniting such diverse conditions under the same umbrella term.

A classic exposition of the naturalist view was developed by Boorse (1976). He defended a value-neutral understanding of illness and health by arguing that illness in general is simply a name for subnormal functioning. He saw disease as an internal state of the organism that interferes with the performance of a natural function of the species (p. 62). Thus, a mental disorder, according to Boorse, is defined as statistically subnormal functioning concerning one or more evolved psychological functions. However, this theory has often been criticized for being too simple and for having too many problems (Bolton, 2008; Brinkmann, 2016).

Bolton (2008) mentions three fundamental problems with Boorse’s naturalist theory: First, the theory simply assumes that deviation from a statistical norm implies problematic functioning, which may – but need not – be the case. Sometimes, functioning below some average is just a difference that does not signify disorder, and sometimes disorders exist as such even if they are quite prevalent (and not statistically deviant) in the population. In psychiatry, this may be the case with depressive disorder, and, in somatic medicine, it may apply to gum disease. These are examples that most people would consider as disorders or diseases, even if they are statistically quite normal, which thus challenges Boorse’s central claim. Second, even if one accepts Boorse’s theory in outline, it is nevertheless unclear where to draw the line between normality and disorder with reference to deviance from a mean (is it one, two or three standard deviations below the mean, for example?). And third, statistical normality is always relative to a population, but the theory does not specify which human population to take as the benchmark relative to which people can be said to suffer from a mental disorder (p. 113).

If Boorse’s theory is an example of a pure naturalist theory, constructionist theories are polar opposites with their emphases on social, cultural, and normative factors. These theories exist in many different forms, but they all agree that one cannot define mental disorders based on alleged natural essences (e.g., natural psychological functions as with Boorse). In that sense, constructionist theories tend to be nominalist and claim that something is a disorder because of social categorizations and not because of anything inherent in the people who suffer. Social constructionist models can be more or less epistemologically and metaphysically radical, but they all “encourage the view that social values and priorities are the sole historical determinants of medicine, music, and marriage”, as Church writes in her account of social constructionist approaches to mental disorder (Church, 2004, p. 393). Some, like Kenneth Gergen, have long advocated for the elimination of what he calls the “deficit discourse” of mental disorder entirely, incarnated in the diagnostic vocabulary (Gergen, 1994), while others, such as the less radical Peter Conrad, argue that diagnosing people with diagnostic categories such as ADHD (which is Conrad’s case) is a species of medicalization that amounts to “social control” (Conrad, 2006). Conrad believes that mental abnormalities, such as the deviance labelled as ADHD, “is not inherent to the individual, the act, or the situation, but [is] rather a process in which certain alleged ‘rule-breaking’ actions come to be defined as deviance” (p. 1). In other words, for a constructionist, there are no mental disorders “out there”, for they are constituted as such by our practices of classification.

There are other more specific approaches that are a bit harder to place on a continuum from naturalist to constructionist or normativist theories. Before moving on to Wakefield’s hybrid theory that distinguishes itself by combining naturalism and normativism, it is worth mentioning neuroscientific and phenomenological theories as two other broad approaches to mental disorder that cut across the naturalist-constructionist continuum. Neuroscientific theories argue, in short, that mental disorders express structural or functional problems in neural processes (Brinkmann et al., 2023). Such theories are quite widespread today, especially after the renaissance of biological psychiatry and the emergence of a human self-interpretation that has been called “the neurochemical self” (Rose, 2007). The recent neuroscientific revolution (if this is the right word) has led to “molecular” interpretations of mental disorders replacing “molar” interpretations, which took the human being as a whole into account (Rose, 2007, p. 199). Pinker captures a general neuroscientific perspective on the mind in his slogan-like insistence that *the mind is what the brain does* (Pinker, 1999). This means that when the brain does something in a dysfunctional way, the mind becomes disordered.

However, a significant problem with the neuroscientific theory is the weak links between the neural and the psychological domains (Brinkmann et al., 2023). Two people, who both suffer from the same mental disorder, may have no brain dysfunction in common, as there is often no one-to-one isomorphism between brain, mind, and behaviour. Furthermore, it is still the case (despite recent technological advances in neuroimagery) that mental disorders are defined and identified phenomenologically and behaviourally. So far, no valid biomarkers have been found in psychiatry (Singh & Rose, 2009). A recent review of the search for biomarkers in psychiatry concluded rather negatively: ”As set out in this review, there are several proteins, metabolites and genes that have been linked with certain neuropsychiatric diseases mainly due to the advance in ‘omics’ technologies. However, none of them have demonstrated to be a real and useful biomarker in clinical practice.” (García-Gutiérrez et al., 2020). For these reasons, the neuroscientific theories are likely bound to remain auxiliary at best, i.e., capable of studying the neural correlates of mental disorders, which is both interesting and important, but something different from studying mental disorders per se (I return to this below).

Finally, we have the phenomenological theories that go back to Jaspers’ classical work (Jaspers, 1997), which stated that mental disorders represent breakdowns in the meaningful connections and relations in our mental lives, but without the theory specifying the causes of such breakdowns (its focus on the experience rather than the underlying cause of the disorder is what makes it phenomenological) (see Brinkmann, 2016). If there is no meaningful connection between what happens and how a person reacts (e.g., between a non-dangerous situation and anxiety), and if the person’s reaction is painful and lingering, then it seems relevant to talk about a mental disorder, according to Jaspers’ phenomenology. A fear of pigeons can be pathological because there is no reason to fear them. A fear of poisonous snakes, on the other hand, may be rational and thus non-pathological, because snakes can in fact be dangerous. So, there is nothing about fear or anxiety in itself that determines its status as pathological. It depends on contextual and relational conditions of meaning. How one sees an instance of fear (and other human actions and reactions), depends on the explanatory and contextual resources that one has available. This opens for a cultural and normative dimension (on which Wakefield expands using the term “harmful”, as we shall see), just as the neuroscientific approach opens for an awareness of dysfunctional mechanisms in the mind or brain (even if these have not been identified through biomarkers), which Wakefield refers to as a dysfunction component. Thus, I now turn to Wakefield hybrid or integrative theory of mental disorder as “harmful dysfunction”. It can be said to integrate both the naturalist and cultural perspectives, and also the neuroscientific and phenomenological ones in a promising way and is therefore worthy of closer scrutiny.

### The Harmful Dysfunction Theory

Jerome Wakefield first published his harmful dysfunction theory of mental disorder in a paper in the early 1990s (Wakefield, 1992). He has since explained, defended, and applied his theory to discussions of different mental disorders, often in collaboration with sociologist Allan Horwitz (e.g., Horwitz & Wakefield, 2007, 2012). He has also critically discussed new and emerging psychiatric diagnoses such as prolonged grief disorder (Wakefield, 2013). His theory of mental illness is called the “harmful dysfunction” theory, since it united these two components in a hybrid understanding of mental disorders. This means that for someone to talk about mental illness/disorder, there should first be something that is *harmful*. A person can only be said to be mentally ill or disordered if that person experiences suffering or distress to a considerable extent. According to Wakefield, this first component is a value component since social norms and values are needed to determine the extent to which something counts as unusual and excessive suffering or distress. How much one should suffer in order for the suffering to be pathological varies across epochs and cultures and is subject to normative assessment. But besides this value component, there is also a purely factual component stemming from the identification of a dysfunction, Wakefield claims. For one is not mentally ill, just because one suffers since suffering can be caused by all kinds of problems and life circumstances. Only if someone’s experience of harm is related to a dysfunction in the person’s mind can the person rightly be said to be mentally ill or disordered.

Wakefield refers to evolutionary psychology, where researchers invoke the existence of genetically based “mental modules” to account for mental functioning (see Brinkmann, in press). These innate psychobiological mechanisms are presented as analogous to physiological mechanisms in bodily organs. And just as a heart can be dysfunctional, when something is wrong with this organ that makes it unable to operate adequately as a blood pump, a mental module can likewise be dysfunctional if it causes a person to feel constant fear, for example, without any dangerous object being present. It is not pathological to feel fear if one is a soldier, who is about to attack the enemy, but if a similar kind of fear is felt in everyday situations that are objectively safe, and if the discomfort is caused by a defect mental module, then we are entitled to talk about a mental disorder, according to Wakefield. In short, for Wakefield, a mental dysfunction is a failure of the capacity of a mental mechanism to perform a function for which it was biologically designed. And when such a dysfunction is coupled with serious harm experienced by the person, then we have a bona fide mental disorder.

A noteworthy consequence of Wakefield’s hybrid theory is that quite a few of psychiatry’s existing diagnoses turn out to be ill founded, because they pathologize ordinary life problems by breaking down the distinction between psychopathology and everyday distress. Horwitz and Wakefield have thus argued that the diagnostic criteria for depression (Horwitz & Wakefield, 2007) and anxiety (Horwitz & Wakefield, 2012) are overinclusive and do not make possible a necessary distinction between common sadness and clinical depression, or between normal fear and pathological anxiety. The reason is that the dysfunction component has not been adequately identified in the psychiatric models that have conflated symptoms with diseases (Nesse, 2020). As psychiatry has moved away from earlier etiological models, a diagnosis is now commonly formulated by psychiatrists by examining the symptoms of patients, and counting them using check lists, and it is has thus become difficult to assess whether the given symptoms are caused by an underlying mental dysfunction or rather by a given life situation. According to Wakefield, sadness should only be diagnosed as depression if there is a dysfunction involved (and not just if the person has been divorced or has suffered a loss), but the existing diagnostic category does not adequately capture such a difference, which has led to overdiagnosis and pathologization of common human sadness and many other forms of misery.

In this way, Wakefield’s hybrid theory of mental disorder as harmful dysfunction has important critical potentials without being anti-psychiatric. It restricts the use of the concept of mental illness significantly without rejecting it. It can serve to warn researchers and practitioners in the psychiatric field of illegitimate pathologizations of ordinary (harmful) experiences and conditions, which are not dysfunctional (and therefore not expressions of mental disorders). A reason to be critical of pathologization is that unpleasant experiences and conditions may arise from social problems such as marginalization, poverty, and social injustice, which run the risk of being interpreted as individual psychopathology when looked at through the diagnostic lens (Brinkmann, 2016). And when the authoritative understanding of an experience or problem employs the psychiatric interpretation (and approaches something as, say, depression), it is of course natural to act as if it were a psychiatric problem (e.g., treating it with anti-depressants). The risk is that pathologization thereby leads to an individualization of social problems and narrows down our ways of understanding and treating the problems that people might otherwise have because of systemic, structural, or political injustice.

### A Dysfunctional Theory?

Despite Wakefield’s important work and his tireless critical efforts, I will now focus on what I see as two key problems in his theory related to the dysfunction component. This component presupposes the existence of discrete mental mechanisms with an evolutionary background that can be separated from cultural-historical processes. My critical argument will center on two ways of problematizing the notion of such mental mechanisms. The first stems from recent critiques of evolutionary psychology that questions the separation of what is naturally evolved from what is culturally acquired (Ingold, 2004, 2011). The second comes from discursive and cultural psychological ideas that simply urge us to reject talk of mental mechanisms as such (e.g., Harré & Moghaddam, 2012).

Since the 1990s, evolutionary psychology has grown to become an influential paradigm in psychology and related disciplines. Evolutionary psychology is of course not alone in endorsing a Darwinian and naturalist outlook on human life, but the paradigm distinguishes itself by its specific theory of the mind. In a founding primer of evolutionary psychology, its originators Cosmides and Tooby (1997) characterized the paradigm in an admirably clear way by outlining its five fundamental principles. Evolutionary psychology is not an area of study in psychology (like vision or cognition), but an approach to psychology as a whole, which they define as: “that branch of biology that studies (1) brains, (2) how brains process information, and (3) how the brain’s information-processing programs generate behavior.” (Cosmides & Tooby, 1997). Again, the mind is what the brain does (Pinker, 1999). The five principles of evolutionary psychology are then outlined as follows: (1) The brain is a physical system that functions as a computer. (2) Our neural circuits were designed by natural selection to solve problems that our ancestors faced during our species’ evolutionary history. (3) Consciousness is just the tip of the iceberg; most of what goes on in your mind is hidden from you. (4) Different neural circuits are specialized for solving different adaptive problems. (5). Our modern skulls therefor house a Stone Age mind. These principles bring together the information processing paradigm in cognitive psychology that emerged after the invention of the digital computer and a broad, Darwinian approach (as discussed in Brinkmann, 2011). The “Stone Age mind” allegedly housed in our skulls is made up by mental modules that have been selected in the evolutionary past and are ultimately constituted by neural circuits. Thus, the mind displays modularity and is a function of the ways in which the brain works. This forms the theoretical background to Wakefield’s understanding of mental mechanisms.

There are good reasons to be skeptical about the principles of evolutionary psychology. Not because of the naturalist and Darwinian assumptions, which most scientists share today, but in a way because they display a poor understanding of Darwin’s naturalism. I have previously argued that we need to save Darwin from evolutionary psychology (see Brinkmann, 2011, on which the following is based), because of critiques raised by biologists and biologically oriented anthropologists. First, it is problematic to claim that neural circuits as such are designed by natural selection, for natural selection does not work on genetic materials. As Ingold has put it: “Natural selection, in short, may occur within evolution, but does not explain it.” (2000, p. 243). The very idea of the genotype, which is necessary to account for the link between gene frequencies and capacities of organisms that are independent of the concrete dynamics of development, is dubious (p. 243). According to Ingold, there simply is no such thing as a genotype conceived as a context-independent design specification (p. 234). It is impossible to factor out what the purely genetically based capacities of humans are (the programs of the “Stone Age mind” that we are allegedly born with), and what is ontogenetically acquired. Genes are parts of long molecules (DNA) that control the production of proteins in organisms, but they interact only with cells that are the building blocks of bodies that operate in environments. Natural selection works on organisms in environments, and not directly on genes. Evolution, according to Ingold’s dynamic view, “is the process in which organisms come into being with their particular forms and capacities and, through their environmentally situated actions, establish the development for their successors.” (Ingold, 1998, p. 95). In this sense, there is already a much closer relationship between biology and culture than evolutionary psychology and Wakefield are aware of.

Natural selection certainly plays a role in the evolutionary process, and it would be foolish to deny that cumulative changes in gene frequencies within populations take place over time, but Ingold’s (2000) point is that there is no link between change in gene frequencies and the capacities of organisms independently of the dynamics of development itself (p. 243). The whole evolutionary-developmental process as such is primary and includes both what we conventionally refer to as natural and cultural aspects (which in itself is a somewhat problematic distinction). The problem with evolutionary psychology in this context is that it is based on the fundamental mistake of assuming that phylogeny is a process separate from ontogeny (Derksen, 2010, p. 480). Natural selection does not “design” organisms, as evolutionary psychologists say, and the role of genes cannot be specified independently of other factors (p. 480).

In short, evolutionary psychology is a problematic approach when it treats the organism and environment as two separate entities, with the environment existing prior to the animal (Withagen & van Wermeskerken, 2010, p. 495). There certainly is a physical world prior to organisms, but no environment exists independently of living organisms. For a physical world becomes an environment (with selection pressures) only when there are animals that operate in it and determine what constitutes the environment. When Darwin studied his earthworms in England, he discovered that they construct their own environments (see Costall, 2004). Or, more precisely, he discovered a mutualism at work in which animals and environments construct each other. The earthworms, which are not well adapted to life in the topsoil (due to their sensitive epidermis that must be kept warm and moist), create vegetable mold in their life processes and thereby transform the econiche in which they live (Withagen & van Wermeskerken, 2010, p. 499). Even such primitive animals construct the very environments in which selection takes place, thus complicating the simple model of natural selection in evolutionary psychology. The offspring of the earthworms inherit not only genes, but also an environment that fits their epidermis better (p. 500). Likewise, birds might have developed color vision because apples are red, which enables the birds to locate and eat the nourishing fruits, and apples might at the same time have developed their red hue because birds have color vision, thereby attracting the birds to help spread the apple seeds. This kind of mutualism works even more pervasively in the case of humans with their immense niche construction capabilities, which – for all we know – were also exercised in the Stone Age (Stotz, 2014).

If such a dynamic and mutualist view is valid, we need to question the very idea of innate mental modules in abstraction from what is “culturally” acquired, and this presents a significant challenge to Wakefield’s dysfunction component as it rests on a belief in such modules. According to Ingold (2004), it makes as little sense to say that the human capacity for walking is innate (e.g., in some alleged “bipedal locomotion module”) as it does to say that cello-playing is. Ingold insists on analyzing all human activities symmetrically. Walking is biological through and through, but also cultural through and through – just like cello-playing and countless other human activities are. We cannot factor out what is biologically hardwired and what is culturally acquired. Walking and cello-playing are both skills that are gradually incorporated (or not) into the operations and life process of human organisms. And people walk differently in different cultures – just as they play music differently. In Japan, people traditionally “walk from the knees”, while Westerners “walk from the hips”, keeping the legs as straight as possible (Ingold, 2004, p. 216). Playing the cello, like walking, is a bodily skill (and no one has to my knowledge claimed the existence of a prespecified mental module or neural circuitry for cello-playing), and cultural differences are not “added on” to biological universals, but are, as Ingold tells us, themselves biological (p. 216).

### Questioning Mentalism

Such arguments should disrupt any hard-and-fast distinctions between “biology” and “culture”, which problematizes Wakefield’s separation of evolutionary based mental mechanisms from their expressions in human lives and cultures. But there is an even more fundamental problem with the notion of mental mechanisms, which has been pointed out for decades by Rom Harré (e.g., 1983, 2002; Harré & Moghaddam, 2012): Namely that they are a scientific illusion. Harré has consistently criticized all forms of mentalism and “mindism” (Brinkmann, 2022), i.e., the view that a special realm of “the mind” understood as mental representations, modules, or mechanisms exist that psychology needs to study in order to understand human doings and sufferings. Just as it is misguided to claim that *the brain* is a subject of psychological functioning (e.g., saying that it perceives, thinks, remembers, or feels), it is in the same way misguided to claim that *the mind* can do these things.

In the case of the brain, Bennett and Hacker (2003) have long criticized what they refer to as the mereological fallacy, which is the error of ascribing properties to a part of something that only makes sense when ascribed to the whole. It is related to what Ryle (1949) classically referred to as category mistakes. The brain is a part of a living organism, and the living organism may certainly perceive something, for example, and although no perception would be possible without a brain, this does not mean that the brain in itself is doing the perceiving. And when the organism is a human person, we may further ascribe higher-order mental predicates to such an entity (a person) and we can then meaningfully talk about a person thinking, remembering, or solving a problem. But the exact same mereological fallacy is very often committed in relation to the mind. Because the word mind is a noun, it is too easy to believe that this noun designates an object and then wonder what properties this object might have. But the mind is not a thing with agentic properties. John Dewey expressed his concerns about this fallacious move many years ago by saying that we should not think about the mind as a noun, but as a verb:

Mind is primarily a verb. It denotes all the ways in which we deal consciously and expressly with the situations in which we find ourselves. Unfortunately, an influential manner of thinking has changed modes of action into an underlying substance that performs the activities in question. It has treated mind as an independent entity, which attends, purposes, cares, and remembers. (Dewey, 1934, p. 268).

This observation is as relevant today as it was in the 1930s, when Dewey wrote it. The mind does not think, feel, or act – only persons can do these things, and talk of the mind is talk about *how* persons act, think, and feel (e.g., intelligently, caringly, angrily etc.).

Just as Wakefield’s harmful dysfunction theory of mental disorder is a hybrid theory, so is Harré’s. For Harré insisted on the need to unify neuroscientific knowledge (about the working of the brain) with an understanding of how people act, think, and feel (Harré, 2002). He developed a sophisticated account of the various vocabularies or “grammars” (in Wittgenstein’s sense) that we employ when talking about persons and their brains: We employ what he called the person grammar when we relate to others as persons. For example, if a person has the right to vote, then it is irreducibly the person, regarded as a citizen, who can put a tick on a ballot paper during general elections, and we cannot meaningfully say that it is the person’s brain, body, or mind that has the right to vote. A person cannot be reduced to anything more elementary, such as a soul, mind, or body, because considered as agents, people cannot be divided into separate parts. In this sense, people are the atoms of social life, and are themselves the source of action – it is not just a part of a person that is acting when someone acts, but the person herself who is acting (see also Sprague, 1999). This also means that it does not make sense to talk about “mental modules” with agentic powers that we can address using the person grammar.

I have already remarked on the difference between persons and organisms, and Harré understood the need for a specific organism grammar, which expresses the norms for how we can meaningfully talk about biological, living beings. The active beings in this case are not people, but organisms. On the one hand, organisms are not exactly like people (it is not the living organism that has the right to vote, but the embodied person), and on the other hand, organisms are also not like things that are driven purely by causal powers. If a stone rolls down a mountainside and starts an avalanche, then this process is normally described in simple causal terms (Brinkmann, 2022). There are already other factors involved if we talk of a mountain goat that runs down the same mountainside looking for a fresh tuft of grass. The stone falls because there is a specific causal process in motion that can be described with reference to the law of gravity. In contrast, the mountain goat runs in order to find some grass. It therefore has a specific organismic goal, and this justifies our description of it as something more than just a thing in motion, which is why we need the organism grammar with a teleological dimension.

Finally, we need a molecule grammar that refers to clusters of molecules as the basic active units. The molecular world is of key importance in different areas of psychology. If a person’s brain is dysfunctional regarding the production of neurotransmitters, then there might be consequences for the person’s psychological capabilities in that they might not be able to think or remember as well (this way of putting it might be simplistic in practice, but it can be assumed for the sake of the argument). Most people make use of the molecule grammar in everyday life, such as when we explain the effects of alcohol on the central nervous system and thus consider its effects on human behavior, which is a legitimate way of describing the process. A person can become drunk after consuming too much alcohol, and, again, it is not the brain or the mind who are drunk, but irreducibly the person – albeit because of molecular processes in the person’s central nervous system. Nevertheless, there are arguably social norms and cultural conventions concerning how to “do drunkenness”, so here – as in many other cases – there is an intricate intersection of different grammars, which I cannot here pursue further. In summary, we can say that the molecule grammar expresses *causal* relations (alcohol can be the cause of diminished mental powers), whereas the organism grammar expresses *functional* relationships. Only the person grammar can be used to express bona fide *normative* relationships that involve reasons for action and responsibility. It also means that in the realm of persons, there are just people´s doings, sufferings, utterances, and experiences. No mechanisms, modules, or representations exist on a “mental” level. Harré and Moghaddam conclude as follows (the P, O, and M domains are short for Person, Organism, and Molecule in the following quote):

There are no hidden mechanisms in the P-domain, according to the point of view being developed here. The program of scientific psychology is not to be fulfilled by postulating an imperceptible realm of unobservable mental mechanisms, as Freud did in introducing the unconscious mind. Scientific ideals in psychology are achieved by making use of the Task/Tool metaphor in proposing neural mechanisms as among the devices that people use for accomplishing their P-grammar tasks. The workings, but not the roles, of these tools are described and explained in the M- and O-grammars. Their domains are tightly woven together in that O-processes are routinely accounted for by recourse to hypotheses about hidden molecular processes. Since at least some M-processes are observable in principle, the proposal of a hidden mechanism explanation can often lead to a research program in an effort to verify the verisimilitude of the working model of that mechanism on which the hypothesis depends. (Harré & Moghaddam, 2012, p. 20)

What they refer to as the Task/Tool metaphor in the quote is simply the observation that the *tasks* carried out by human beings (remembering something, finding one’s way, solving a problem, passing an exam, getting married, etc.) are always identified based on the person grammar, whereas the functioning of the *tools* employed (the brain, neurotransmitters, but also materials tools as parts of social practices) is described based on organism and molecule grammars. Identifying the tasks should be primary in psychology, for it makes no sense to scan a person’s brain to understand the neural correlate of reading, for example, if one has not already identified what reading is as a normative task carried out by persons. So, in a sense Harré does find a place for mechanisms, but only on the “tool” (e.g., molecule) level, and not on the person or “task” level.

### Beyond Mental Mechanisms

After the above account of Wakefield’s harmful dysfunction theory of mental disorder and the ensuing critique of its idea of mental modules, it is now time to ask if Wakefield’s theory remains relevant. The key question is if the dysfunction component necessarily needs to be constituted by mental mechanisms, or if a dysfunction can be located and defined in other ways? Of course, one consequence of the problematization of the whole idea of an evolved set of mental mechanisms in abstraction from the life processes of persons could be simply to discard the whole harmful dysfunction theory. As I understand it, this is Bolton’s (2008) conclusion, when he states that “the current science has tended to break down the sharp divide between what is natural, evolved, and what is social, then the rationale for naturalist definitions of mental disorder is also broken down.” (p. 152). And he includes Wakefield’s theories among the naturalist ones that are, allegedly, faulty.

However, this conclusion might be too hasty, for if we follow Harré’s analysis, we can say that even if the natural-social (or natural-cultural) divide is problematic, we still need to separate person talk from molecule and organism talk. This does not necessarily mean that persons are uniquely “cultural”, and molecules/organisms uniquely “natural”, for, bearing Ingold’s arguments in mind, persons and their abilities are at once cultural and natural through and through, just as their brains and bodies are at once cultural and natural. So, the interesting distinction in this context is not the one between what is natural and what is cultural, but rather between different aspects of “naturecultural” lives, some of which can be described as the actions and experiences of persons, while others are better described as causal workings of the brain and other organs when persons carry out different tasks.

I borrow the term “natureculture” from Joseph Rouse’s (2023) recent book in which he brings naturalist and evolutionary perspectives together with a normativist one, not with a specific focus on mental disorder, however, but as a general framework for the social sciences. His main point is that social practices – with all their norms and affordances that provide reasons for action for human beings – should be seen as forms of biological niche construction. Like the earthworms live in topsoil, which they co-produce through their life activities, so human lives are conducted in and through social practices that are at once material and normative, natural and cultural – hence the naturecultural perspective. My argument will then be to say that in order to understand human lives in naturecultural practices, we need not invoke talk of mentalist modules or mechanisms but can follow Harré in using the three vocabularies (person, organism, molecule) to refer to various aspects of these social practices or econiches (see also Rose et al., 2022).

A naturecultural theory of mental disorder could thus take the following lines: If persons are not able to participate adequately (which is, of course, a judgment informed by naturecultural norms) in social practices, this can be because of unrealistic demands, social exclusion, oppression, marginalization, or other factors that we normally think about as political or systemic. In this case, it seems illegitimate to pathologize the individual for her inadequacies, and instead a fair and humane response would be to change the environment through collective action. But inadequate participation may also result from problems carried by the individual due to dysfunctional molecular or organismic processes that are likely to remain problematic even if the individual were to live under different environmental conditions. In Harré’s terminology, it might be relevant to talk about the “tools” being damaged, thereby making it difficult for the individual person to adequately carry out her “tasks” relative to the naturecultural norms of social practices. In this way, Wakefield’s theory of harmful dysfunction could remain relevant after the critique of mentalism, i.e., if the dysfunction component is seen in light of dysfunctional tools on molecular or organismic levels rather than residing in some problematic mental mechanism. When understood in this way, it becomes relevant to address a continuum of people’s problems related to dysfunctions at one end and related to environmental challenges on the other, and so it might not always be easy to determine if someone has a mental disorder or not. This is no reason for abandoning the concept but should alert us to the fact that sometimes it is preferable to help people adapt to their circumstances, e.g., by developing tools (in a broad sense) with which to cope, and sometimes it is better to change the circumstances, and one cannot know which one is better without looking closely at the concrete case.

There is a further challenge to the harmful dysfunction theory that deserves to be mentioned here. It comes from those increasingly influential naturalist perspectives that focus on mismatches between biopsychological functions developed in prehistoric environments and their current manifestations in a modern world, which might create problems for individuals (Swanepoel et al., 2017). Here the argument is that a condition such as ADHD was once just a normal form of exploratory behavior involving a broad form of attention, which was adaptive in the so-called environment of evolutionary adaptedness (EEA). Today, however, this way of being human becomes problematic in the highly constrained environment we see in contemporary schools for example. If so, there is no dysfunction involved (but only harm given the fact that the environment does not match the individual’s capabilities), and Wakefield seemingly cannot account for why such conditions are mental disorders. He can, however, reply that this simply represents an illegitimate pathologization of a variation of human existence. In any case, however, if the problematization of evolved mental mechanisms is a challenge to Wakefield’s theory (for the reasons discussed above), then it is likewise a challenge to such mismatch theories. For how can it be helpful to talk about a default way of being human based on the EEA, now that we know so much about neural plasticity, mutualism, and dynamic relationships between nature, culture, and social life? In this light, it seems that although mismatch theories conceptualize disorders in a different way than Wakefield (viz., as mismatches rather than dysfunctions), they are challenged by the same counterarguments invoked in the present article as Wakefield’s harmful dysfunction theory.

However, there are other ways of conceiving of mismatches. Within an evolutionary approach, Garson (2021) suggests that some mental disorders are a result of developmental plasticity rather than dysfunction in Wakefield’s sense (of a defective evolved mechanism). This could lead us to an understanding of the dysfunction component in an alternative way, which is something that should be explored if one is skeptical of the legacy from evolutionary psychology. Garson’s idea does not rest on an idea of a mismatch between an evolutionary past and modern society, but rather concerns what he calls a developmental mismatch. This is not a mismatch between life in the EEA (as programmed in mental modules) and modern society, but rather between an individual’s life in a “formative environment” (which includes both prenatal conditions and the postnatal environment) and one’s later environment, e.g., as an adult. It thus restricts itself to ontogeny. Garson does not claim that this applies to all mental disorders, but it seems to be an interesting hypothesis that some forms of anxiety or conduct disorder, for example, that emerged as responses to help protect someone as a child, can become less helpful later in life and thus present themselves as mental disorders. Again, Wakefield will likely respond that this represents a pathologization of unwanted behaviors rather than mental disorders, but if one’s brain and central nervous system (the “tools” as Harré would say) have developed in ways that are both dysfunctional and harmful later in life due to naturecultural plasticity, then it seems that we have a candidate for mental disorder that satisfies Wakefield’s criteria, even in the absence of a defective mental module.

## Conclusions

I this article, I have examined the harmful dysfunction theory of mental disorder, developed by Jerome Wakefield. I find this theory interesting and valuable, because of its attempt find a non-arbitrary demarcation of mental disorder from common human suffering. I certainly believe that we as human beings have reasons to alleviate the suffering of others in any form it might take, but it is important to figure out if someone’s suffering is the result of a challenging situation or rather a dysfunction. Wakefield´s critique of psychiatry states that this distinction is often blurred, and people’s sufferings are thus often pathologized and individualized.

However, if there are problems with Wakefield’s dysfunction component that come from evolutionary psychology, as I have argued, then we need to explore if there are other ways of thinking about dysfunction if we want to preserve the main tenets of a harmful dysfunction theory. This is what I have attempted in the present article, and it led me to a naturecultural understanding of persons with significant inspiration from Harré. Another promising avenue relates to what is now known as the 4E approach to mental life. The four Es mean that mental life is conceived as embodied, embedded, enacted, and extended (although not all scholars in the field subscribe to all of these). Recently, Rose (2023) has added a fifth E, viz., the *experience* of people in distress. Hoffman (2016), Krueger (2022), Nielsen (2022), and Sneddon (2002) are examples of scholars who have applied the 4E approach to mental illness and disorder specifically, but they do so without specifying what they mean by the concept of mental disorder. I thus believe that the ambition of the present paper to bring Wakefield’s theory into the discussion, and possibly form an alliance with the non-mentalist approach of the 4E and 5E schools, points forward in a promising way. I hope the discussion will continue, and I am encouraged to believe that there is great potential in theorizing mental disorder in ways that are not simply naturalist and essentialist or cultural and normativist but finds a way to integrate these perspectives. A naturecultural approach can be a first step, and we need to move on with further reflections and also empirical studies to inform and challenge our current conceptions if we are truly to understand and help people who suffer.

## Data Availability

No datasets were generated or analysed during the current study.

## Footnotes

1 There is a relevant discussion about differences between talk of mental *illness* and *disorder*, but in the context of this article, I use them interchangeably. Normally, we would say that a *person* is ill, if he or she suffers from a disorder in the mind or the body.
